# The Dresden Surgical Anatomy Dataset for Abdominal Organ Segmentation in Surgical Data Science

**DOI:** 10.1038/s41597-022-01719-2

**Published:** 2023-01-12

**Authors:** Matthias Carstens, Franziska M. Rinner, Sebastian Bodenstedt, Alexander C. Jenke, Jürgen Weitz, Marius Distler, Stefanie Speidel, Fiona R. Kolbinger

**Affiliations:** 1 Department of Visceral, Thoracic and Vascular Surgery, University Hospital and Faculty of Medicine Carl Gustav Carus, Technische Universität Dresden, Dresden, Germany; 2 Division of Translational Surgical Oncology, National Center for Tumor Diseases (NCT/UCC) Dresden, Dresden, Germany; 3 Centre for Tactile Internet with Human-in-the-Loop (CeTI), Technische Universität Dresden, Dresden, Germany; 4 Else Kröner Fresenius Center for Digital Health (EKFZ), Technische Universität Dresden, Dresden, Germany

**Keywords:** Gastrointestinal system, Endoscopy, Translational research

## Abstract

Laparoscopy is an imaging technique that enables minimally-invasive procedures in various medical disciplines including abdominal surgery, gynaecology and urology. To date, publicly available laparoscopic image datasets are mostly limited to general classifications of data, semantic segmentations of surgical instruments and low-volume weak annotations of specific abdominal organs. The *Dresden Surgical Anatomy Dataset* provides semantic segmentations of eight abdominal organs (colon, liver, pancreas, small intestine, spleen, stomach, ureter, vesicular glands), the abdominal wall and two vessel structures (inferior mesenteric artery, intestinal veins) in laparoscopic view. In total, this dataset comprises 13195 laparoscopic images. For each anatomical structure, we provide over a thousand images with pixel-wise segmentations. Annotations comprise semantic segmentations of single organs and one multi-organ-segmentation dataset including segments for all eleven anatomical structures. Moreover, we provide weak annotations of organ presence for every single image. This dataset markedly expands the horizon for surgical data science applications of computer vision in laparoscopic surgery and could thereby contribute to a reduction of risks and faster translation of Artificial Intelligence into surgical practice.

## Abstract (summary)

**Table:** 

Measurement(s)	Laparoscopic Surgery
Technology Type(s)	Laparoscopy
Factor Type(s)	Presence and location of anatomical structures within laparoscopic images
Sample Characteristic - Organism	Homo sapiens
Sample Characteristic - Environment	abdomen
Sample Characteristic - Location	abdominal cavity

## Background & Summary

Laparoscopic surgery is a commonly used technique that facilitates minimally-invasive surgical procedures as well as robot-assisted surgery and entails several advantages over open surgery: reduced length of hospital stay, less blood loss, more rapid recovery, better surgical vision and, especially for robotic procedures, more intuitive and precise control of surgical instruments^1,2^. Meanwhile, a lot of the information in the image is not used, because the human attention is not able to process this immense amount of information in real time. Moreover, anatomical knowledge and medical experience are required to interpret the images. This barrier represents a promising starting point for the development of Artificial Intelligence (AI)-based computer-based assistance functions.

The rapidly developing methods and techniques provided by the usage of AI, more precisely the automated recognition of instruments, organs and other anatomical structures in laparoscopic images or videos, have the potential to make surgical procedures safer and less time-consuming^3–6^. Open-source laparoscopic image datasets are limited, and existing datasets such as Cholec80^7^, LapGyn4^8^, SurgAI^9^ or the Heidelberg Colorectal Data Set^10^ mostly comprise image-level annotations that allow the user to differentiate whether or not the structure of interest is shown in an image without giving information about its specific spatial location and appearance. However, such pixel-wise annotations are required for a variety of machine learning tasks for image recognition in the context of surgical data science^11^. In a clinical setting, such algorithms could facilitate context-dependent recognition and thereby protection of vulnerable anatomical structures, ultimately aiming at increased surgical safety and prevention of complications.

One major bottleneck in the development and clinical application of such AI-based assistance functions is the availability of annotated laparoscopic image data. To meet this challenge, we provide semantic segmentations that provide information about the position of a specific structure by annotations of each pixel of an image. Based on video data from 32 robot-assisted rectal resections or extirpations, this dataset offers a total amount of 13195 extensively annotated laparoscopic images displaying different intraabdominal organs (colon, liver, pancreas, small intestine, spleen, stomach, ureter, vesicular glands) and anatomical structures (abdominal wall, inferior mesenteric artery, intestinal veins). For a realistic representation of common laparoscopic obstacles, it features various levels of organ visibility including small or partly covered organ parts, motion artefacts, inhomogeneous lighting and smoke or blood in the field of view. Additionally, the dataset contains weak labels of organ visibility for each individual image.

Adding anatomical knowledge to laparoscopic data, this dataset bridges a major gap in the field of surgical data science and is intended to serve as a basis for a variety of machine learning tasks in the context of image recognition-based surgical assistance functions. Potential applications include the development of smart assistance systems through automated segmentation tasks, the establishment of unsupervised learning methods, or registration of preoperative imaging data (e.g. CT, MRI) with laparoscopic images for surgical navigation.

## Methods

This dataset comprises annotations of eleven major abdominal anatomical structures: abdominal wall, colon, intestinal vessels (inferior mesenteric artery and inferior mesenteric vein with their subsidiary vessels), liver, pancreas, small intestine, spleen, stomach, ureter and vesicular glands.

### Video recording

Between February 2019 and February 2021, video data from a total of 32 robot-assisted anterior rectal resections or rectal extirpations performed at the University Hospital Carl Gustav Carus Dresden was gathered and contributed to this dataset. The majority of patients (26/32) were male, the overall average age was 63 years and the mean body mass index (BMI) was 26.75 kg/m^2^ (Table 1). All included patients had a clinical indication for the surgical procedure. Surgeries were performed using a standard Da Vinci® Xi/X Endoscope with Camera (8 mm diameter, 30° angle, *Intuitive Surgical*, Item code 470057) and recorded using the *CAST-System* (*Orpheus Medical GmbH*, Frankfurt a.M., Germany). Each record was saved at a resolution of 1920 × 1080 pixels in MPEG-4 format and lasts between about two and ten hours. The local Institutional Review Board (ethics committee at the Technical University Dresden) reviewed and approved this study (approval number: BO-EK-137042018). The trial, for which this dataset was acquired, was registered on clinicaltrials.gov (trial registration ID: NCT05268432). Written informed consent to laparoscopic image data acquisition, data annotation, data analysis, and anonymized data publication was obtained from all participants. Before publication, all data was anonymized according to the general data protection regulation of the European Union.

**Table 1:** Patient characteristics.

**Age** (years)	63 ± 9
**BMI** (kg/m^2^)	26.8 ± 3.0
**Sex**	
Female	6 (18.8%)
Male	26 (81.3%)
**Indication**	
Rectal cancer	31 (96.9%)
Other	1 (3.1%)
**Distance from anocutaneous line**	
<6 cm	14 (43.8%)
6–11 cm	13 (40.6%)
≥12 cm	5 (15.6%)
**Surgical resection technique**	
LAR, TME/PME	19 (59.4%)
ISR, TME	5 (15.6%)
APR, TME	3 (9.4%)
AR, PME	5 (15.6%)
**UICC stage**	
0	1 (3.1%)
I	10 (31.3%)
IIA	5 (15.6%)
III	1 (3.1%)
IIIA	2 (6.3%)
IIIB	8 (25.0%)
IIIC	2 (6.3%)
IVA	3 (9.4%)

For age and BMI, mean ± standard deviation are displayed. For all other data, the table gives total numbers and proportions of the entire cohort (32 patients). Abbreviations: abdominoperineal resection (APR), anterior resection (AR), body mass index (BMI), intersphincteric resection (ISR), low anterior resection (LAR), partial mesorectal excision (PME), total mesorectal excision (TME), Union for International Cancer Control (UICC).

### Temporal annotation of videos

The surgical process was temporally annotated by one medical student with two years of experience in robot-assisted rectal surgery (MC, FMR) using b<>com *Surgery Workflow Toolbox* [Annotate] version 2.2.0 (*b<*>*com*, Cesson-Sévigné, France), either during the surgery or retrospectively, according to a previously created annotation protocol (Supplementary File 1), paying particular interest to the visibility of the abovementioned anatomical structures. Ubiquitous organs (abdominal wall, colon and small intestine), intestinal vessels, and vesicular glands were not specifically annotated temporally.

### Frame extraction

To achieve a highly diverse dataset, videos from at least 20 different surgeries were considered for each anatomical structure. From each considered surgical video, up to 100 equidistant frames were randomly selected from the total amount of video data displaying a specific organ. As a result, this dataset contains at least 1000 annotated images from at least 20 different patients for each organ or anatomical structure. The number of images extracted and annotated per organ and surgery as well as the number of segments and the mean proportions of non-segmented background per organ are listed in Table 2.

**Table 2:** Distribution of images in the dataset per organ and surgery.

Surgery index	Abdominal wall	Colon	Inferior mesenteric artery	Intestinal veins	Liver	Pancreas	Small intestine	Spleen	Stomach	Ureter	Vesicular glands	Total
**01**	—	—	—	—	83	80	—	5	—	83	—	**251**
**02**	—	—	—	—	64	55	—	—	70	52	—	**241**
**03**	63	57	61	61	90	61	56	64	84	85	55	**737**
**04**	59	66	56	—	36	82	51	75	52	54	69	**600**
**05**	61	58	—	49	14	74	58	91	58	55	56	**574**
**06**	59	53	44	—	—	—	57	—	—	—	54	**267**
**07**	61	52	58	58	—	42	57	—	44	71	—	**443**
**08**	—	—	—	—	—	52	—	28	18	—	—	**98**
**09**	68	56	57	58	—	—	55	—	—	—	—	**294**
**10**	58	61	—	40	—	—	60	—	43	—	61	**323**
**11**	56	68	56	56	40	64	58	71	87	58	64	**678**
**12**	64	58	56	46	5	61	57	85	84	90	55	**661**
**13**	—	—	—	—	83	—	—	81	—	—	62	**226**
**14**	56	68	50	52	—	—	51	54	—	—	55	**386**
**15**	—	—		—	80	—	—	72	—	—	—	**152**
**16**	58	64	57	55	—	9	60	66	45	43	47	**504**
**17**	53	57	56	57	—	81	57	—	77	48	59	**545**
**18**	61	59	58	60	44	64	57	—	78	65	59	**605**
**19**	58	55	51	42	—	—	53	—	—	62	56	**377**
**20**	51	62	53	56	42	91	56	45	86	66	66	**674**
**21**	52	57	54	59	47	88	50	50	44	75	72	**648**
**22**	59	63	56	59	9	56	60	64	79	70	—	**575**
**23**	53	63	—	49	—	—	58	83	78	—	58	**442**
**24**	52	63	57	64	8	32	52	74	41	25	70	**538**
**25**	54	54	56	50	56	—	51	—	63	—	55	**439**
**26**	50	56	57	52	10	28	54	78	74	71	63	**593**
**27**	—	—	—	—	—	51	—	14	57	—	—	**122**
**28**	—	58	42	—	83	—	—	—	—	72	—	**255**
**29**	—	66	55	44	62	51	—	—	71	—	62	**411**
**30**	—	—	—	—	86	—	—	—	31	57	—	**174**
**31**	—	—	—	—	81	51	—	16	66	73	—	**287**
**32**	—	—	—	—	—	—	—	75		—	—	**75**
**Total**	**1206**	**1374**	**1090**	**1067**	**1023**	**1173**	**1168**	**1191**	**1430**	**1275**	**1198**	**13195**
Median number of segments (range)	1 (1–7)	2 (1–9)	1 (1–5)	1 (1–6)	1 (1–5)	3 (1–10)	2 (1–7)	1 (1–4)	1 (1–5)	1 (1–4)	1 (1–9)	
Mean % background (SD)	74% (14%)	88% (7%)	98% (2%)	99% (1%)	80% (18%)	97% (3%)	84% (10%)	97% (4%)	95% (6%)	99% (1%)	97% (4%)	

For each surgery and organ, the total number of images in the dataset is given. Moreover, this table summarizes the median number of segments and the mean proportion of non-segmented background per organ. Abbreviation: standard deviation (SD).

For anatomical structures without a temporal annotation (abdominal wall, colon, intestinal vessels, small intestine and vesicular glands), sequences displaying the specific organ were selected and merged manually using *LossLessCut* version 3.20.1 (developed by Mikael Finstad). Random frames were extracted from the merged video file using a *Python* script (see section “Code availability”). The extraction rate (extracted frames per second) was adjusted depending on the duration of the merged video to extract up to 100 images per organ per surgery. Images were stored in PNG format at a resolution of 1920 × 1080 pixels.

For liver, pancreas, spleen, stomach and ureter, temporal annotations served as a basis for the frame-extraction process using the abovementioned *Python* script. Based on a TXT file with temporal annotations of organ presence, equidistant frames were extracted from respective sequences for each organ as outlined above.

The resulting frames were audited and images that were not usable (e.g. the organ is not visible because it is concealed completely by an instrument, the complete field of view is filled with smoke, severely limited visibility due to a blurred camera) were excluded manually.

No automated filtering processes were applied to specifically select or avoid images (e.g. based on mutual information). To maintain the variability inherent to intraoperative imaging, no image preprocessing steps such as adaptation of image intensity or contrast, or window size) were performed. Images were directly extracted from the videos recorded during surgery, converted into PNG (lossless). These images were then directly annotated.

**Fig. 1:**
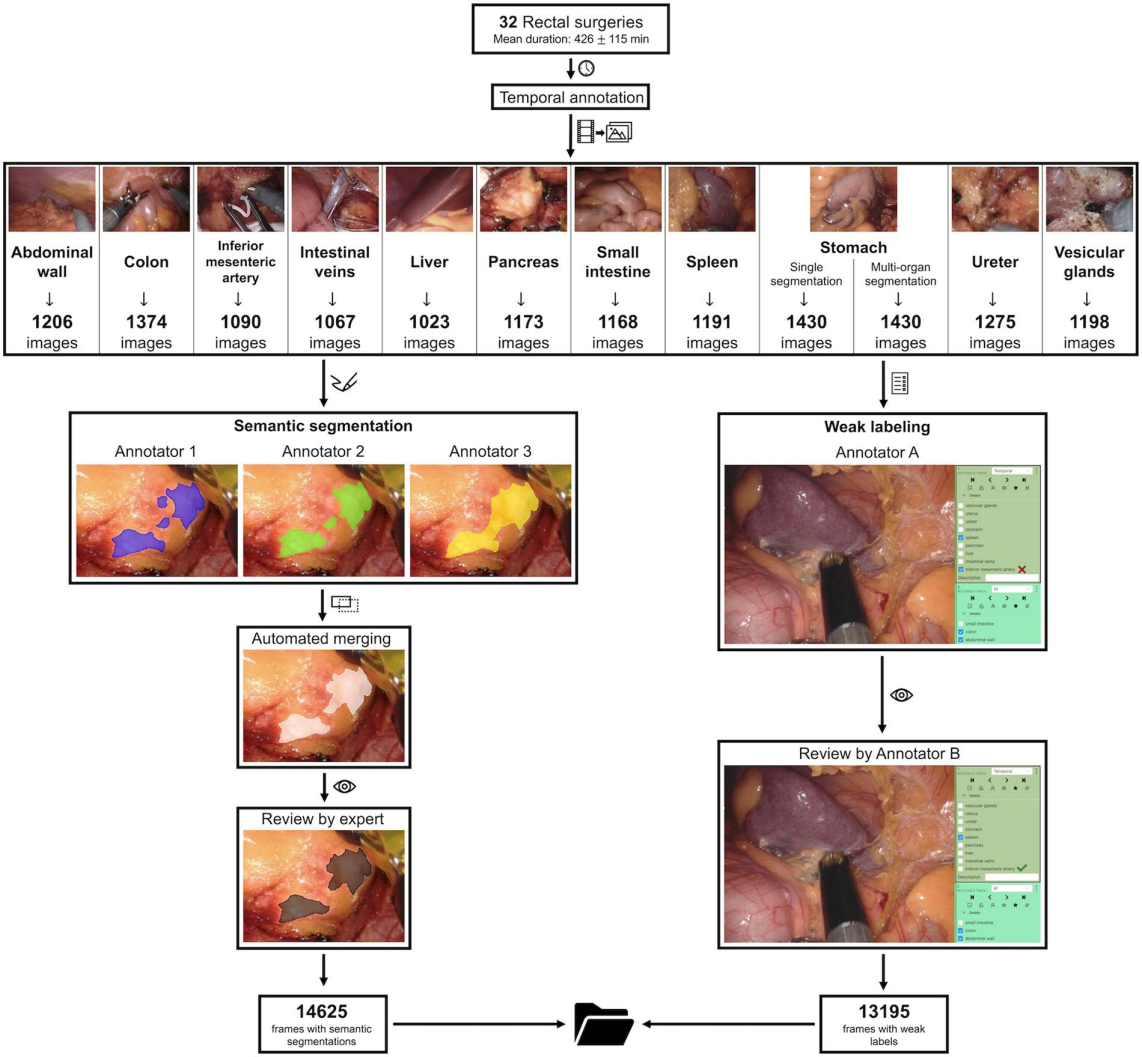
Overview of the data acquisition and validation process. Based on temporal annotations of 32 rectal resections, three independent annotators semantically segmented every single image with regard to the pixel-wise location of the respective organ. These segmentations were merged and individual segmentations were reviewed alongside the merged segmentation by a physician with considerable experience in minimally-invasive surgery, resulting in the final pixel-wise segmentation (left panel). Moreover, every single image was classified with regard to the visibility of all individual anatomical structures of interest by one annotator and independently reviewed (right panel).

The resulting dataset includes over 1000 images from at least 20 surgeries for each anatomical structure (Fig. 1).

### Semantic segmentation

**Fig. 2:**
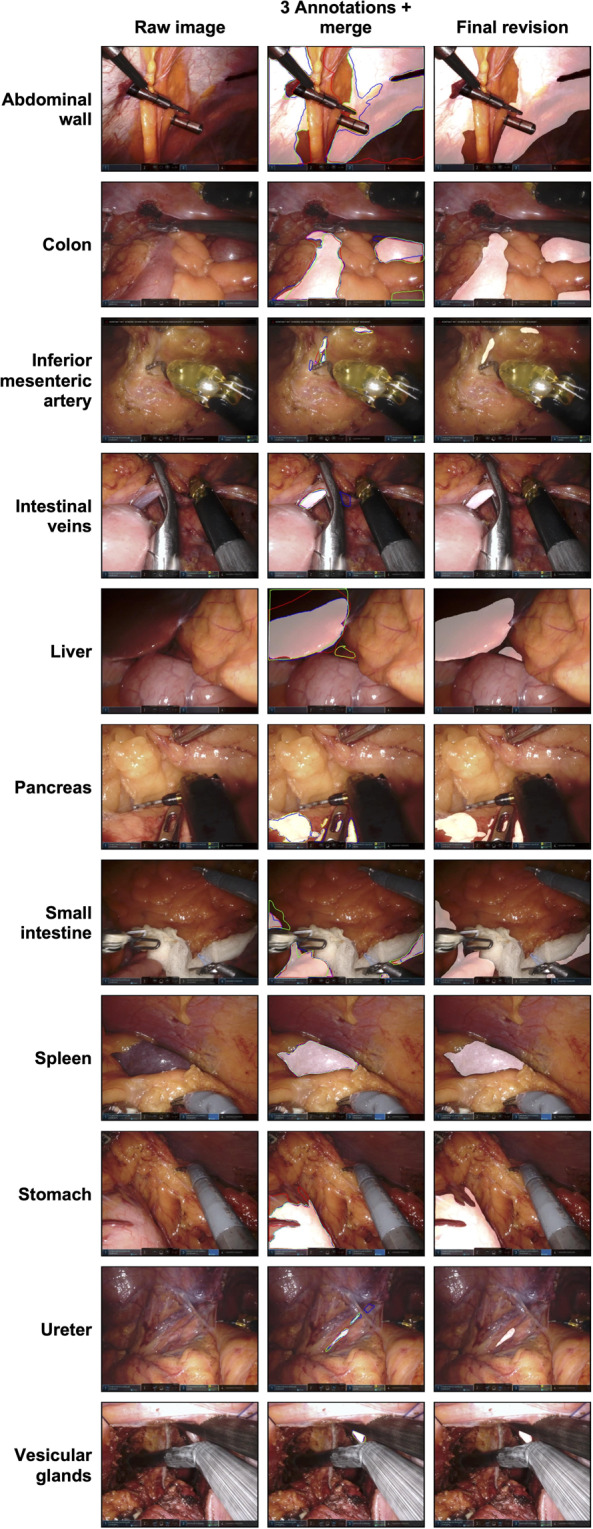
Sample images of each anatomical structure. The figure displays a raw image (left column), the three pixel-wise annotations and the merged annotation (middle column) as well as the final reviewed segmentation (right column). The three annotations are shown as red, green and blue lines. The merged version and the final reviewed segmentation are displayed as white transparent surfaces.

For pixel-wise segmentation, we used *3D Slicer* 4.11.20200930 (https://www.slicer.org) including the *SlicerRT* extension, an open-source medical image analysis software^12^. The anatomical structures were manually semantically segmented with the *Segment Editor* function using a stylus guided tablet computer running *Microsoft Windows*. The settings made during segmentation were “scissors”, operation “fill inside”, shape “free form”, slice cut “symmetric”. As a guideline we generated a segmentation protocol that describes inclusion criteria for each considered anatomical structure in detail (Supplementary File 2). Each individual image was semantically annotated according to this guideline by three medical students with basic experience in minimally-invasive surgery. Thus, exactly one specific anatomical structure was finally segmented in each image (e.g. the colon was pixel-wise annotated in each of the 1374 colon images). In addition, one multi-organ-segmentation dataset was created out of the 1430 stomach frames. The stomach dataset was chosen for this purpose because these images very often show various organs, such as the colon, small intestine or spleen as well as the abdominal wall. Subsequently, the three individual annotations were automatically merged (see section “Code availability”). Individual annotations alongside merged segments were reviewed and adjusted by a physician with three years of experience in minimally-invasive surgery. Figure 1 gives an overview over the image generation and verification process. Example annotations are provided in Fig. 2.

### Weak labeling

Weak labels provide information about the visibility of different anatomical structures in the entire image. Weak labels were annotated by one medical student with basic experience in minimally-invasive surgery and reviewed by a second one in each frame (Fig. 1).

The complete dataset is accessible at figshare^13^.

## Data Records

The Dresden Surgical Anatomy Dataset is stored at figshare^13^. Users can access the dataset without prior registration. The data is organized in a 3-level folder structure. The first level is composed of twelve subfolders, one for each organ/anatomical structure (*abdominal_wall, colon, inferior_mesenteric_artery, intestinal_veins, liver, pancreas, small_intestine, spleen, stomach, ureter and vesicular_glands*) and one for the multi-organ dataset (*multilabel*).

Each folder contains 20 to 23 subfolders for the different surgeries that the images have been extracted from. The subfolder nomenclature is derived from the individual index number of each surgery. Each of these folders contains two versions of 5 to 91 PNG-files, one raw image that has been extracted from the surgery video file and one image that contains the mask of the expert-reviewed semantic segmentation (black = background, white = segmentation). The raw images are named *imagenumber.png*, (e.g. *image23.png*), the masks are named *masknumber.png* (e.g. *mask23.png*). In the *multilabel* folder there are separate masks for each of the considered structures visible on the individual image (e.g. *masknumber_stomach.png*). The image indices always match for associated images.

Each surgery- and organ-specific folder furthermore contains a CSV file named *weak_labels.csv* that contains all information about the visibility of the eleven regarded organs in the respective images. The columns in these CSV files are ordered alphabetically: Abdominal wall, colon, inferior mesenteric artery, intestinal veins, liver, pancreas, small intestine, spleen, stomach, ureter and vesicular glands.

Additionally, the folders *anno_1, anno_2, anno_3* and *merged* can be accessed from the surgery- and organ-specific subfolders. These folders contain the masks generated by the different annotators and the automatically generated merged version of the masks, each in PNG format.

## Technical Validation

To merge the annotations of the three different annotations for each image in the dataset, the STAPLE algorithm^14^, which is commonly used for merging different segmentations in biomedical problems, was applied. Each annotator received the same weight. The merged annotations were then, together with the original segmentations of the annotators, uploaded to a segmentation and annotation platform called *CVAT* (https://github.com/openvinotoolkit/cvat)^15^ hosted at the National Center for Tumor Diseases (NCT/UCC) Dresden. The physician in charge of reviewing the data could then log-in, select the most appropriate annotations for each image and, if necessary, adjust them.

To evaluate the extent of agreement between the segmentations of the individual annotators and the merged annotation with the final annotation of each image, we computed two standard metrics for segmentation comparison^16^:

- F1 score, which showcases the overlap of different annotation with a value of 0 to 1 (0: no overlap, 1: complete overlap)
- Hausdorff distance, a distance metric, which calculates the maximum distance between a reference annotation and another segmentation. Here we have normalized the Hausdorff distance via the image diagonal, resulting in values between 0 and 1, which 0 indicates that there is no separation between the two segmentations and 1 meaning there is a maximum distance between the two.

The results of this comparison can be found in Table 3, sorted according to the different tissue types. The table shows that for most organs there is no large discrepancy between the merged annotations and the final product, with most F1 scores being over 0.9 indicating a large overlap and the low value for the Hausdorff distance indicating that no tendencies for over or under-segmentation were present. Only the F1 score for the ureter class seems to indicate that the expert annotator had to regularly intervene, though the difference still seems to be minimal as indicated by the low Hausdorff distance.

**Table 3:** Comparison of the different annotators and their merged annotation to the final annotation.

Organ	A1	A2	A3	A4	A5	A6	Merged
**Abdominal Wall**	0.91 (0.10)	—	—	0.96 (0.04)	0.91 (0.09)	—	0.97 (0.03)
**Colon**	—	0.92 (0.04)	0.88 (0.10)	0.97 (0.02)	0.93 (0.04)	0.92 (0.05)	0.96 (0.03)
**Inferior mesenteric artery**	0.62 (0.10)	—	—	0.91 (0.03)	0.87 (0.04)	—	0.91 (0.03)
**Intestinal veins**	0.80 (0.05)	—	—	0.93 (0.03)	0.84 (0.04)	—	0.91 (0.03)
**Liver**	0.91 (0.06)	0.93 (0.03)	0.98 (0.01)	—	—	—	0.94 (0.04)
**Pancreas**	0.80 (0.04)	—	0.95 (0.01)	0.95 (0.01)	—	—	0.98 (0.01)
**Small intestine**	0.92 (0.05)	—	—	0.99 (0.01)	0.95 (0.03)	—	0.98 (0.02)
**Spleen**	—	—	0.97 (0.01)	0.97 (0.01)	0.92 (0.02)	—	1 (0)
**Stomach**	—	—	0.95 (0.02)	0.92 (0.04)	0.93 (0.03)	—	0.99 (0.01)
**Ureter**	0.77 (0.03)	—	0.88 (0.02)	—	0.85 (0.02)	—	0.87 (0.03)
**Vesicular glands**	0.84 (0.03)	—	—	0.95 (0.01)	0.92 (0.01)	—	0.94 (0.01)
**Multi label**	0.95 (0.03)	—	0.96 (0.04)	—	0.73 (0.22)	—	0.92 (0.06)

Each cell contains the F1 score and the normalized Hausdorff distance in parentheses.

Most annotators also seemed to regularly agree with the final annotation, though not always with the same degree as the merged annotation, justifying the fusion via STAPLE. Similar to the merged annotations, there were larger discrepancies in regard to the ureter class. Generally though, at least two annotators seemed to largely agree with the expert annotations.

## Usage Notes

The provided dataset is publicly available for non-commercial usage under the Creative Commons Attribution CC-BY. If readers wish to use or reference this dataset, they should cite this paper.

The dataset can be used for various purposes in the field of machine learning. On the one hand, it can be used as a source of further image material in combination with other, already existing datasets. On the other hand, it can be used to create organ detection algorithms working either with weak labels or with semantic segmentation masks, for example as a basis for further development of assistance applications^17^. Proposed training-validation-test splits as well as results of detailed segmentation studies are reported in a separate publication ^18^.

## Supplementary information

Supplementary File 1

Supplementary File 2

## Data Availability

The scripts for frame extraction, annotation merging, and statistical analysis, as well as the results of the statistical analysis are made public on https://gitlab.com/nct_tso_public/dsad and via https://zenodo.org/record/6958337#.YvIsP3ZBxaQ. All code is written in *python3* and freely accessible.
