# Supplementary material for: The Dresden Surgical Anatomy Dataset for Abdominal Organ Segmentation in Surgical Data Science
Source: Sci Data. 2023 Jan 12;10:3. doi: 10.1038/s41597-022-01719-2 (PMC9837071; doi:10.1038/s41597-022-01719-2)
Supplement: Supplementary file 1 [file 41597_2022_1719_MOESM1_ESM.docx]

**The Dresden Surgical Anatomy Dataset for abdominal organ**

**segmentation in surgical data science**

**Supplementary File 1: Annotation guideline for temporal annotation of robot-assisted rectal resections**

The following section lists the phase and step divisions as well as typical actions that occur during annotation. Further actions are possible.

**Phase-independent actions:**

- assistant - general action - pull - small bowel/colon/rectum/mesocolon/Gerota’s fascia/… - medial/cranial/lateral/…
- assistant - general action - clean - endoscope
- assistant - general action - suck - blood
- surgeon - visibility 1/2 - stomach
- surgeon - visibility 1/2 - spleen
- surgeon - visibility 1/2 - liver
- surgeon - visibility 1/2 - pancreas
- surgeon - visibility 1/2 - ureter
- surgeon - visibility 1/2 - vesicular gland

**Phase: Preparation and intraabdominal orientation**

**Step: Trocar placement**

- surgeon - general action - insert - trocar

**Step: Intraabdominal orientation**

- surgeon - general action - inspect - abdominal cavity
- surgeon - general action - separate - adhesion
- surgeon - general action - move - small bowel

**Phase: Mobilization of colon (medial)**

**Step: Peritoneal Incision (medial)**

- surgeon - general action - incise - peritoneum - medial

**Step: Preparation/Clipping/Dissection of Inferior Mesenteric Artery**

- surgeon - general action - dissect - Inferior Mesenteric Artery
- surgeon - general action - expose - Inferior Mesenteric Artery
- surgeon - general action - separate - mesocolon, Gerota’s fascia
- assistant - general action - clip - Inferior Mesenteric Artery
- assistant - general action - cut - Inferior Mesenteric Artery

**Step: Separation of mesocolon and Gerota’s fascia (medial)**

- surgeon - general action - separate - mesocolon, Gerota’s fascia

**Step: Preparation/Clipping/Dissection of Inferior Mesenteric Vein**

- surgeon - general action - dissect - Inferior Mesenteric Vein
- surgeon - general action - expose - Inferior Mesenteric Vein
- surgeon - general action - separate - mesocolon, Gerota’s fascia
- assistant - general action - clip - Inferior Mesenteric Vein
- assistant - general action - cut - Inferior Mesenteric Vein

**Step: Separation of mesocolon and Gerota’s fascia (medial)**

- surgeon - general action - separate - mesocolon, Gerota’s fascia

**Phase: Mobilization of colon (lateral)**

**Step: Peritoneal Incision (lateral)**

- surgeon - general action - separate - adhesion
- surgeon - general action - incise - peritoneum - lateral

**Step: Separation of mesocolon and Gerota’s fascia (lateral)**

- surgeon - general action - separate - mesocolon, Gerota’s fascia
- surgeon - general action - dissect - greater omentum

**Phase: Total mesorectal excision and dissection of rectum**

**Step: Peritoneal incision superior to peritoneal fold**

- surgeon - general action - tie - rectum
- surgeon - general action - separate - mesorectum, Denonvillier’s fascia
- surgeon - general action - separate - mesorectum, Waldeyer’s fascia

**Step: High mesorectal dissection**

- surgeon - general action - dissect - mesorectum

**Step: Low mesorectal dissection**

- surgeon - general action - separate - mesorectum - Denonvillier’s fascia
- surgeon - general action - separate - mesorectum - Waldeyer’s fascia

**Step: Linear stapling of rectum**

- surgeon - general action - staple - rectum
- assistant - general action - rinse - water

**Phase: Extraabdominal preparation of anastomosis**

**Step: Pfannenstiel incision**

**Step: Open preparation and dissection of the descending colon**

**Step: Placement of circular stapler head**

**Step: Transverse coloplasty**

**Step: Temporary closure of the Pfannenstiel incision**

**Phase: Intraabdominal preparation of anastomosis**

**Step: Circular stapling of rectum**

**Step: Water Probe**

**Phase: Ileostomy**

**Phase: Closure**
