# Supplementary material for: The Dresden Surgical Anatomy Dataset for Abdominal Organ Segmentation in Surgical Data Science
Source: Sci Data. 2023 Jan 12;10:3. doi: 10.1038/s41597-022-01719-2 (PMC9837071; doi:10.1038/s41597-022-01719-2)
Supplement: Supplementary file 2 [file 41597_2022_1719_MOESM2_ESM.docx]

**The Dresden Surgical Anatomy Dataset for abdominal organ**

**segmentation in surgical data science**

**Supplementary File 2: Guideline for the segmentation of anatomical structures**

**General:**

- the respective structures are visible on every single image of the dataset assigned to them; the degree of recognizability may vary
- mark only intact, uninjured and clearly assignable wall/parenchyma of the structures
- avoid marking
  - areas substantially covered with smoke/blood/fat
  - areas not properly visible due to a dirty camera
  - large alimentary vessels (small vessels should be marked)
  - instruments/compresses/threads/…
  - anything outside the image margins
- mark whenever it is possible to recognize the structure in an area that is
  - dark
  - slightly covered with smoke/blood/fat/fascia
  - small: sometimes, parts of the structure are visible in tiny areas, e.g. in instrument slots
- note that a structure may be visible in several small areas in a single image

**Abdominal wall:**

- abdominal wall is mostly shown in the upper half of the image
- only mark fatty tissue that is located within the abdominal wall layers (i.e., if there is peritoneum above it); omit fat appendages on the surface of the abdominal wall
- please pay close attention to the borders of the abdominal wall segment if the colon is still attached to the abdominal wall
- folds of the abdominal wall, e.g. the plica umbilicalis, should be marked

**Colon:**

- risk of confusion with small intestine: colon has appendices epiploicae, haustra, taeniae
- colon is often attached to the abdominal wall in the images
- avoid marking areas with fat appendages, for example appendices epiploicae or remnants after the separation of the colon and the greater omentum

**Inferior mesenteric artery:**

- typical color: white to light pink, often small alimentary vessels are visible
- risk of confusion with veins: veins are usually more bluish in color and appear softer
- in case of doubt it can be helpful to identify the artery at the moment of clipping in order to recognize it at an earlier stage

**Intestinal veins:**

- bluish appearance
- in some cases, larger veins can be seen that are located retroperitoneally and therefore do not belong to the "intestinal veins" (e.g. the testicular vein)
- risk of confusion with arteries: arteries usually have a whiter color and appear stiffer
- in case of doubt it can be helpful to identify the vein at the moment of clipping in order to recognize it at an earlier stage

**Liver:**

- varying structure possible (cirrhosis, metastases, …)
- avoid marking the gallbladder
- if the liver or its margins are in the shadow, marking is demanding, but there is almost always a line visible where the liver ends

**Pancreas:**

- typical structure: white to light pink, lobular structure in surrounding adipose tissue
- it’s almost always necessary to mark several spots

**Small intestine:**

- risk of confusion with colon:
  - small bowel usually is narrower, lighter in color and smoother than large intestine
  - does not have appendices epiploicae, haustra, taeniae
  - peristalsis can resemble haustra

**Spleen:**

- if it is located within a fluid accumulation, special attention is required when determining the margins

**Stomach:**

- distinction against colon: the stomach is
  - lighter in color,
  - has no taeniae, haustra, appendices epiploicae
- distinction against gallbladder: stomach has no tight connection to the liver
- avoid marking large alimentary vessels (small vessels should be marked)

**Ureter:**

- sometimes peristaltic movement can be visible on the images
- vascular pattern on the ureter can help in retrieval
- risk of confusion with the tendon of the iliopsoas muscle: tendon is whiter, more iridescent, more fibrous and usually lies on dark red muscle
- risk of confusion with arteries: often distinguishable by the localization and clipping of the artery in the course; ureter appears softer and is usually whiter

**Vesicular glands:**

- color: white to pink
- when the exposure is being started, sometimes only very small areas are visible, you can skip a few images ahead to detect the correct area
